# Supplementary material for: Systematic and quantitative view of the antiviral arsenal of prokaryotes
Source: Nat Commun. 2022 May 10;13:2561. doi: 10.1038/s41467-022-30269-9 (PMC9090908; doi:10.1038/s41467-022-30269-9)

**Supplementary Information**

**Supplementary Figure 1: Hit score repartition for HMM of single-gene systems.**

Each graph represents the distribution of hit scores (GA) for the HMM profile of each single-gene system. Bars represented in red have a score below the threshold and are not detected by DefenseFinder. Blue bars have a score above the threshold and are detected by DefenseFinder. Single-gene systems are detected using only one HMM and the specificity could not be increased with colocalization with another protein. Thus, we used those graphs to set up specific GA cut to avoid over detection.


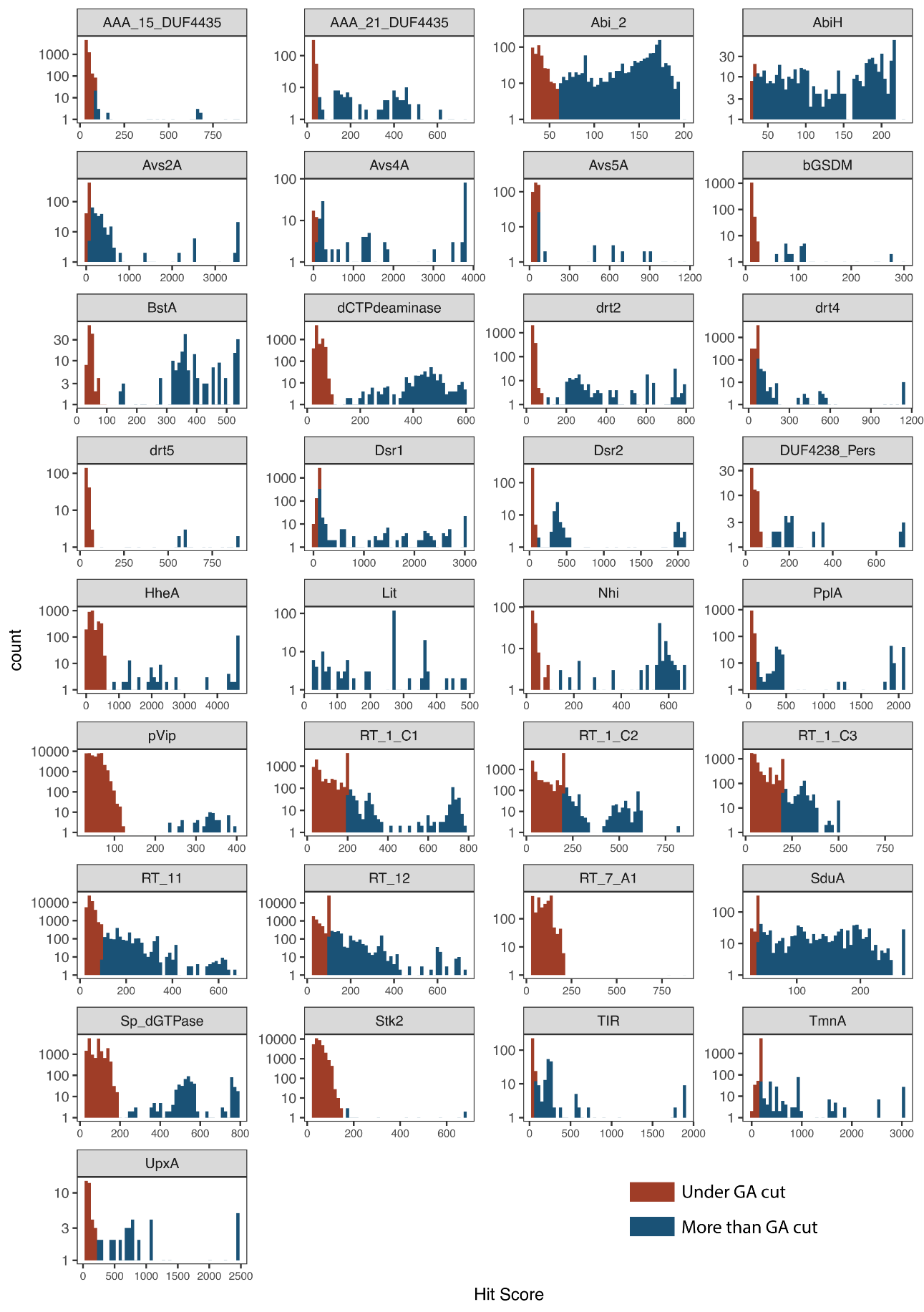


**Supplementary Figure 2: Evaluation of GA threshold for Doron’s system proteins using ROC-curves**

For each GA threshold, True positive rate (TPR) (or sensitivity) corresponds the ratio TP/(TP+FN), where True Positives (TP) are the proteins found by DefenseFinder among proteins annotated by Doron and colleagues’ paper. False Negatives (FN) are proteins that were not detected by DefenseFinder among the same set of proteins. The TPR is thus the proportion of protein from Doron et al. that are found by DefenseFinder. For the False Positives Rate (FPR), we ran DefenseFinder on a set of genomes where the system was not detected by Doron and colleagues. For every system except Shedu, we used about 3000 genomes. For Shedu, 6000 genomes were used. FPR for each GA threshold was calculated as FP/MaxFP. False positives (FP) are the proteins wrongly annotated by DefenseFinder at a given GA cut and the maximum false positive (MaxFP) is the number of false positives without GA cut. Lowering the cut GA increases the TPR, but with a higher FPR. The arrows show the GA threshold used for each protein and the GA used in DefenseFinder is written in parentheses. To estimate the number of false positive systems, we consider that the presence of a given protein follows a Poisson distribution. The probability of a false system is the probability that all the proteins required for a system to be detected are present within 10kb for a pair. The MacSyFinder rules use a distance parameter of 5 proteins, which gives us a conservative interval of 10kb, assuming 2 kb for the average protein size. The Estimated FP systems represent the estimated number of systems found if th e proteins were randomly distributed in the genomes in the used database (around 3000 genomes and 12.5 Gb). Finally, the observed false positive systems are indicated.


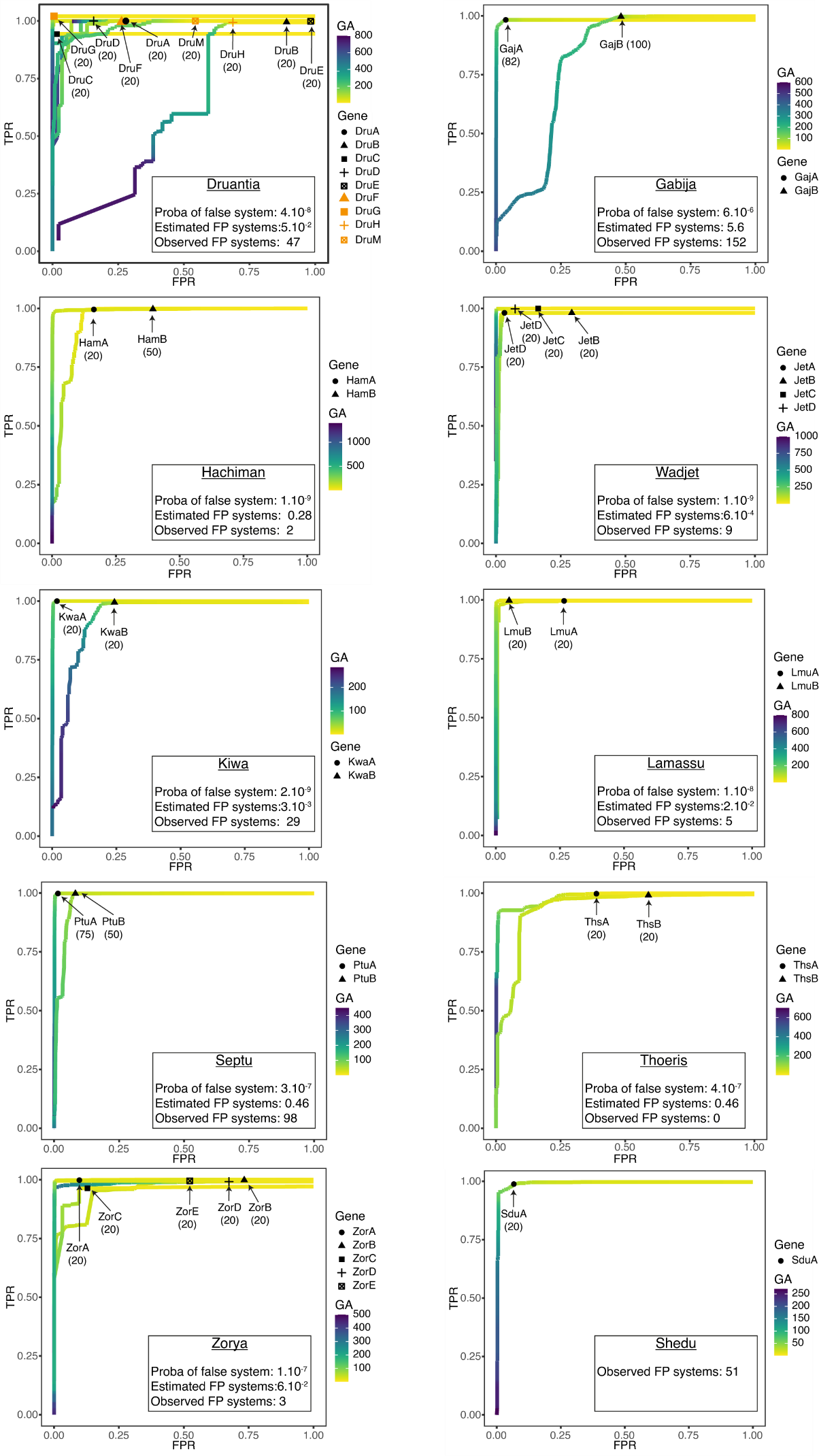


**Supplementary Figure 3: Validation of DefenseFinder models**

**a.** Analysis of true and false positives for Lamassu’s proteins depending on the hit score. True positive rates were computed as DefenseFinder detection on protein from (Doron et al 2018) divided by the number of proteins detected by Doron and colleagues. False positives were calculated by running DefenseFinder on genomes where Doron’s and colleagues have not found any system. Normalized false positive rate is computed as FP/MaxFP, where False positive (FP) is the number of off-target proteins at each GA cut and Maximum false positive (MaxFP) is the number of off-target proteins without any GA cut. The continuous line counts every false hit protein whereas discontinuous lines represent false positives that are present with other proteins of the system. The vertical bar represents the GA cut that was chosen for DefenseFinder. **b.** Percentage of genomes with R-M subsystems found by DefenseFinder and previous detection by (Oliveira et al., 2014). The observed difference is linked to less strict detection in profiles (coverage and GA cut). However, for Type I RM systems, we found the subunit S in 96,8 % of the previously undetected cases, suggesting these are real RM. Furthermore, the HMM profiles used for the 2014 study do not detect a part of the current REBASE database that evolved since 2013. **c.** Percentage of genomes with the different systems and subsystems detected by DefenseFinder and previous detections. Druantia, Gabija, Hachiman, Kiwa, Lamassu, Septu, Shedu, Thoeris, Wadjet and Zorya were found in (Doron et al., 2018); AVAST, DRT, Dsr, Gao’s and RADAR in (Gao et al., 2020); BREX in (Goldfarb et al., 2015); CBASS in (Millman et al., 2020); dCTPdeaminase and dGTPase in (Tal et al., bioRxiv 2021); DISARM in (Ofir et al., 2018); GasderMIN in (Johnson et al., 2021); Retron in (Millman et al., 2020); Viperin in (Bernheim et al., 2021). Differences for Gabija and Septu could be linked to subclasses of Septu and Gabija that are found with the same genetic architecture but was not found in previous detections. For Gao_Iet, we do not have access to detailed detection so we cannot properly determine the reason for inconsistency between our detection and the one performed in Gao’s et al. For SspBCDE, comparison of results with Wang et al., 2021 demonstrates a sensitivity of 97,9%, however only 477 systems were detected on RefSeq complete genome database, which could be explained by the difference in the database species distribution. **d.** System sensibility and specificity for Doron’s systems, CBASS and DISARM. Sensibility was calculated using genomes where the system was found and specificity where no systems were found in previous detection. Low specificity for Septu and Gabija is linked to detection of proteins slightly different from previous detections but with the same domains. **e.** Protein sensitivity on the REBASE database for each R-M protein.


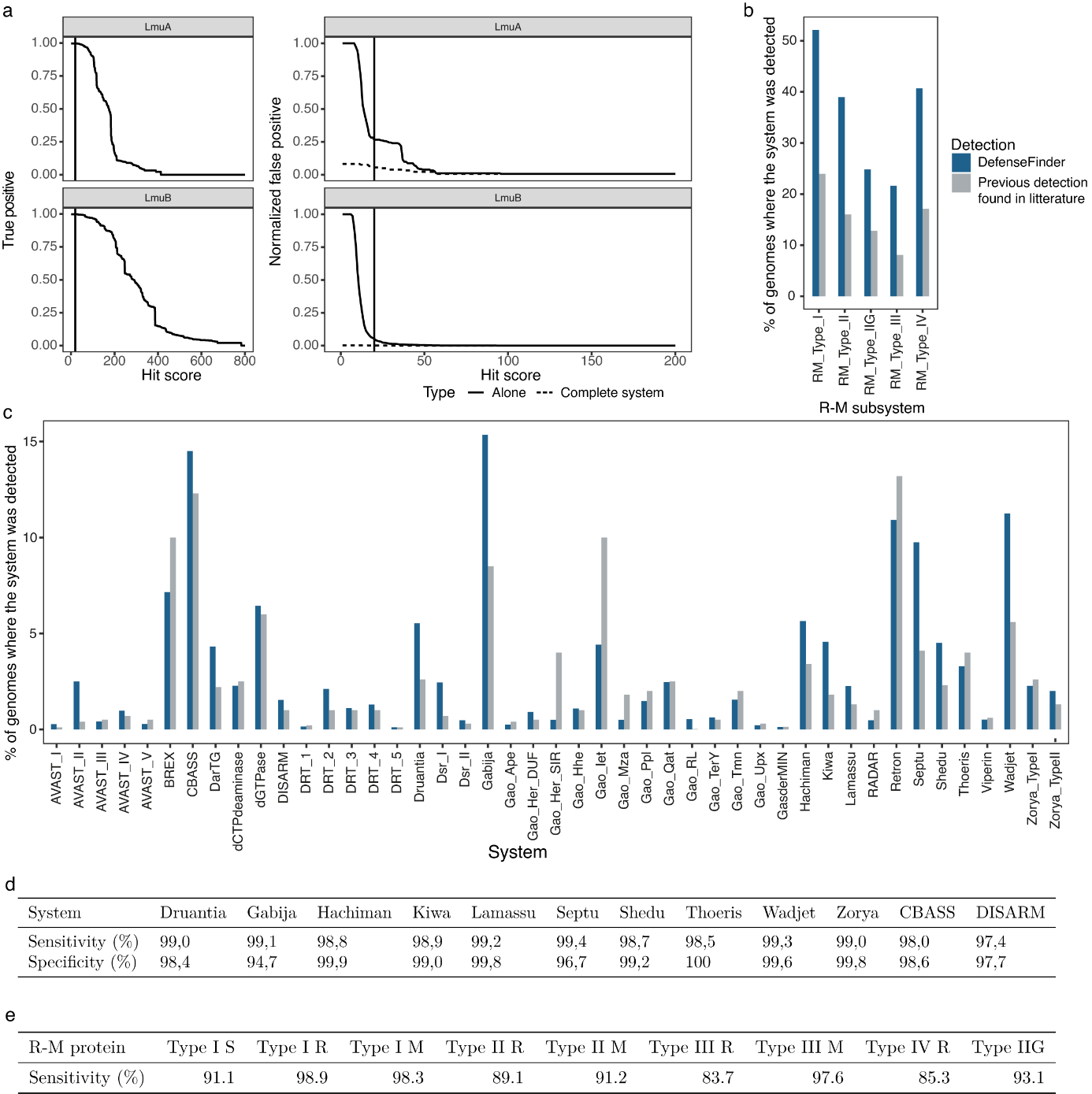


**Supplementary Figure 4: Detection comparison between DefenseFinder and PADLOC.** The y axis represents systems searched by PADLOC and DefenseFinder. The analysis was made with 18 683 genomes. For each genome we look if each system was detected by PADLOC or DefenseFinder. The x-axis represents the proportion of detection by PADLOC, DefenseFinder or both for all genomes where the system is found. The detection difference for Septu can be explained by a subclass of Septu found by DefenseFinder.


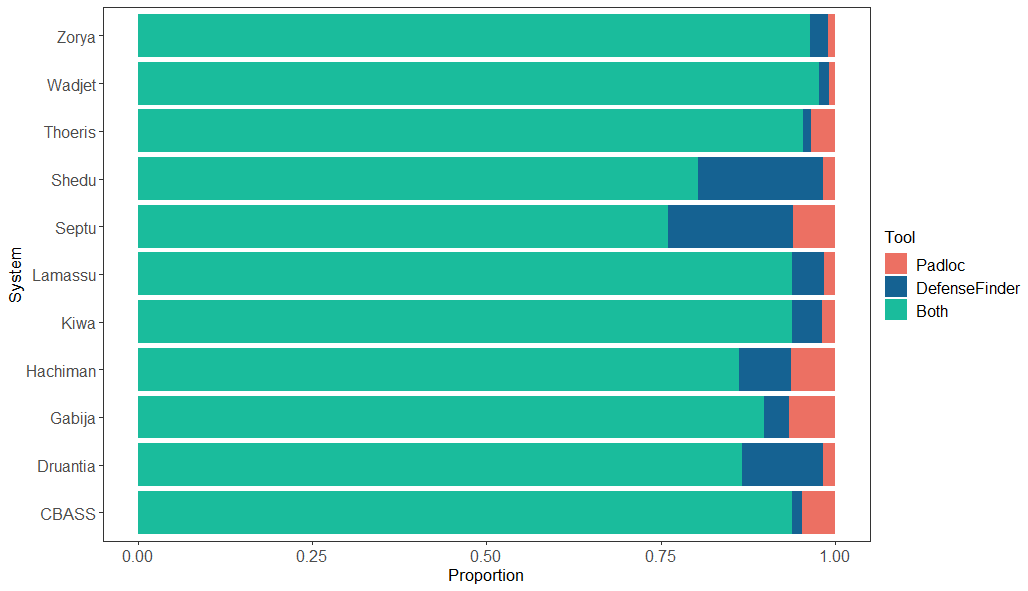


**Supplementary figure 5: Families of antiviral systems are correlated with the total number of antiviral systems**

a. Distribution of the number of families antiviral systems per genome. The x-axis was cut at 20 for data visualization purposes.  **b.** Correlation between the families of antiviral systems and the total number of antiviral systems (Spearman ρ=0.79 P-value<0.0001).


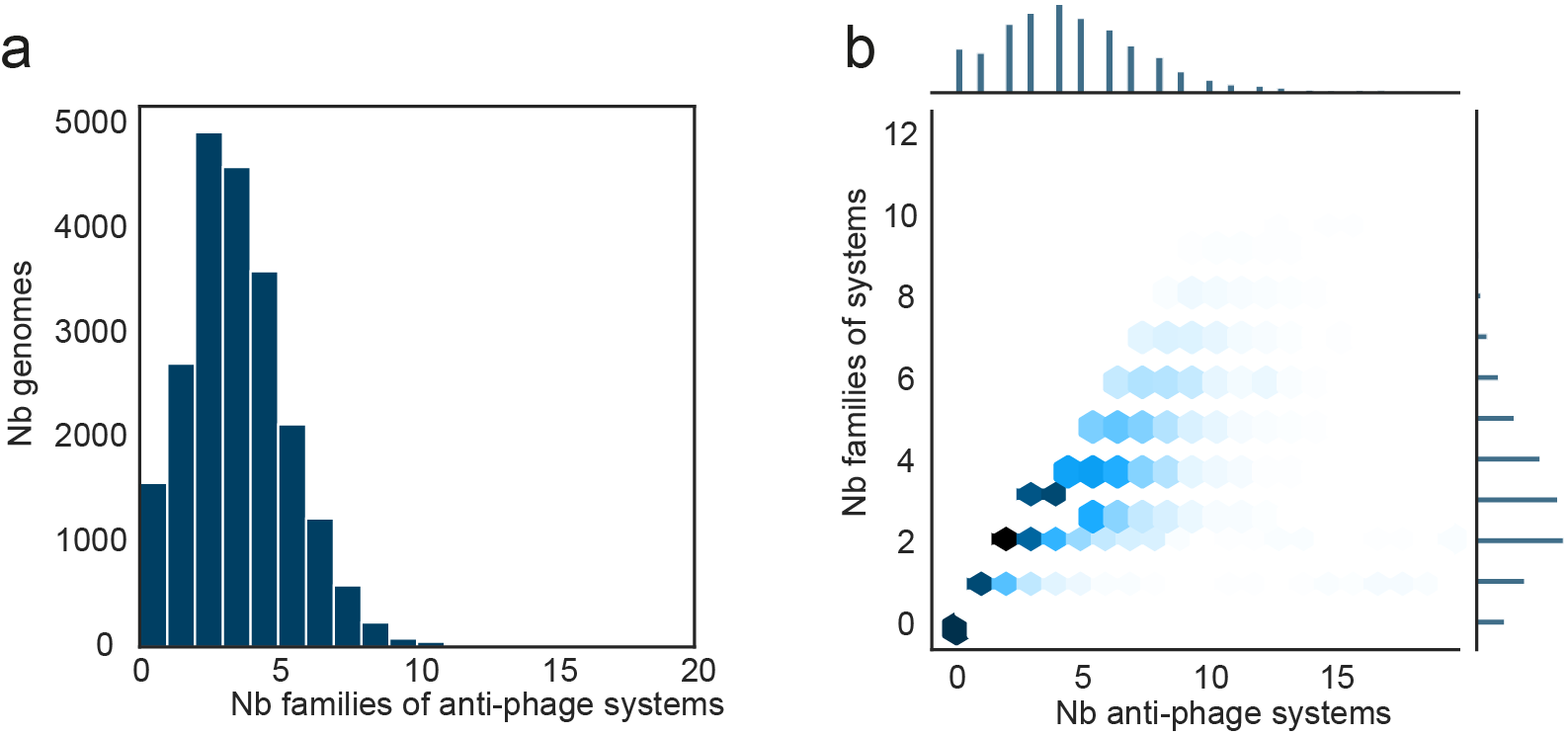


**Supplementary figure 6: Prophage detection**

**a.** Distribution of the number of prophages per genome.  **b.** Correlation between number of prophages and genome size.


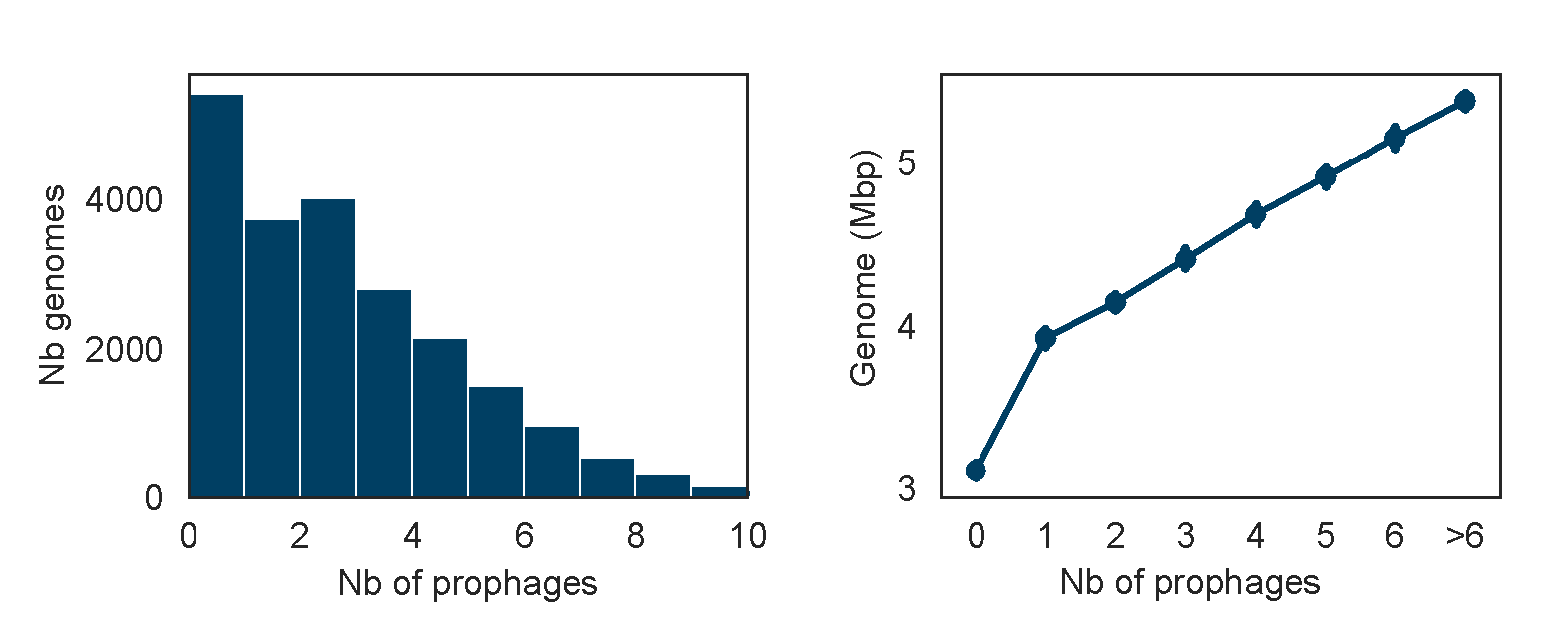


**Supplementary Figure 7: Distribution of antiviral systems in genomes without RM**

**a.** Distribution of anti-viral systems.  **b.** Distribution per phyla**
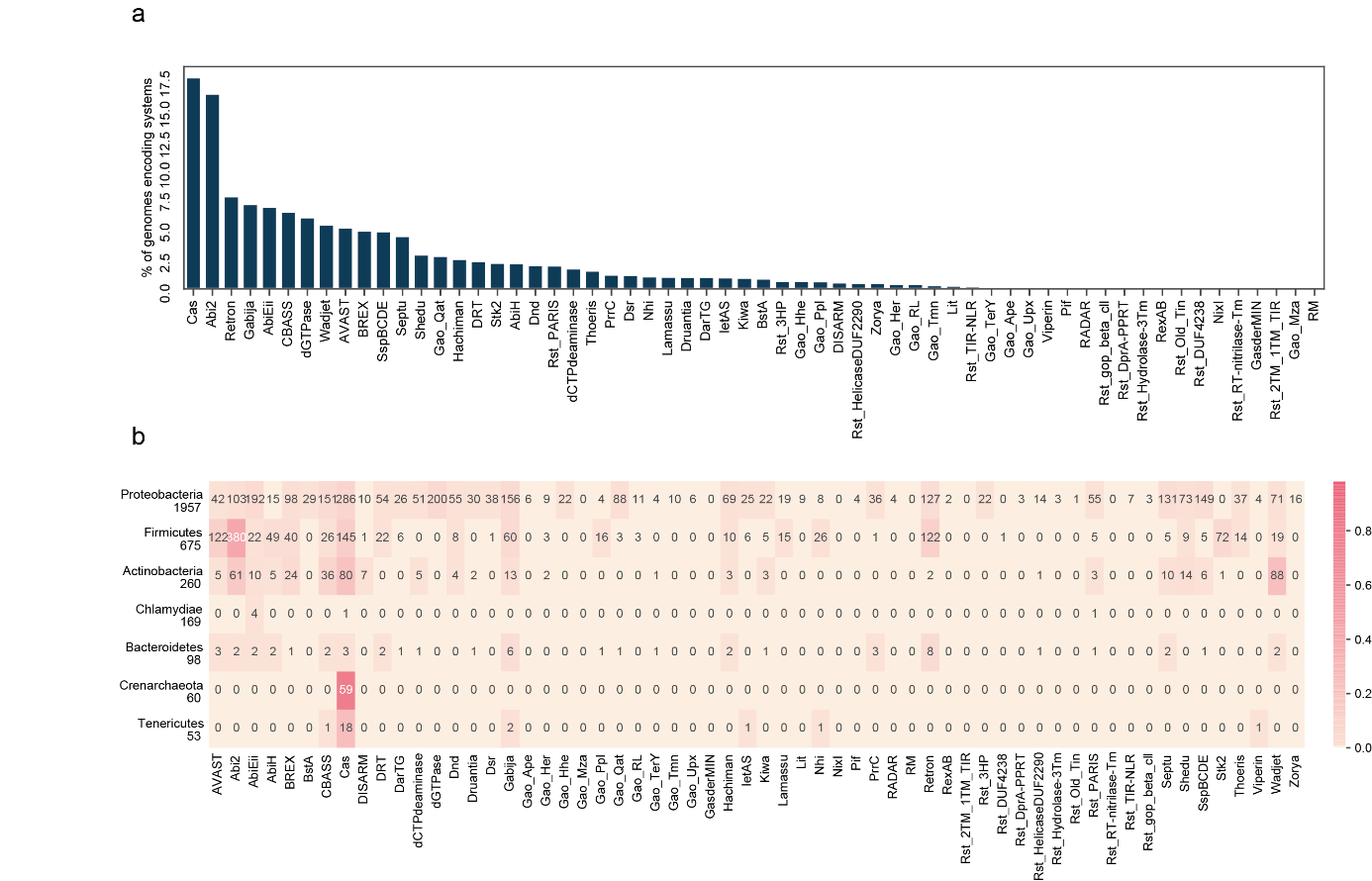
**

**Supplementary Figure 8: Anti-viral arsenal of diverse bacterial species**

Each panel shows the distribution of the total number of systems in the species (top panel), the frequency of the 20 most common antiviral systems in prokaryotes in this species (bottom panel) and a phylogenetic tree of the species with the presence/absence of the 10 most common antiviral systems in prokaryotes.


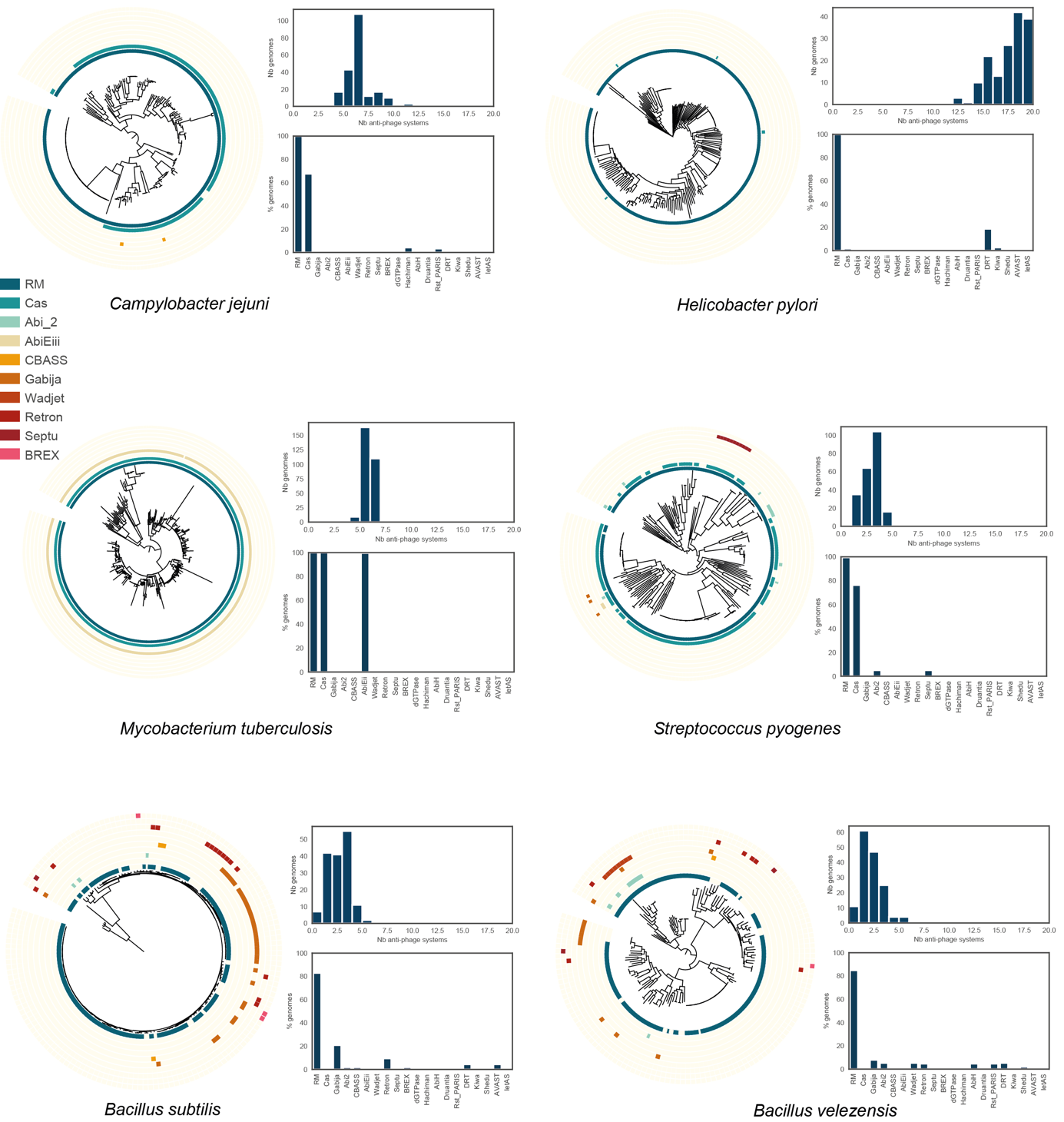


**
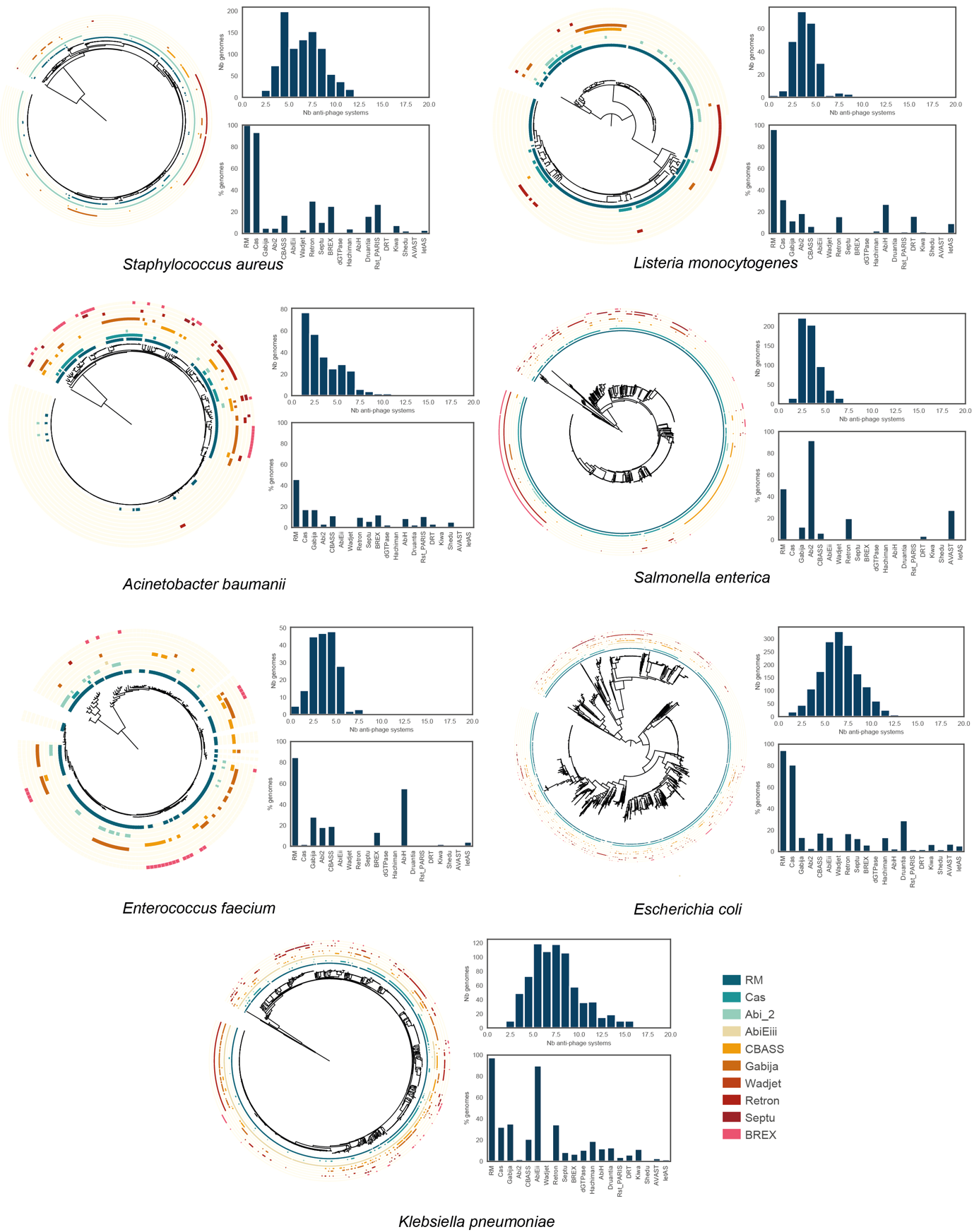
**

**Supplementary Figure 9: Anti-viral arsenal of bacterial species are diverse**

**a.** Scatter plot for the anti-viral arsenal of prokaryotes. **b.** Correlation between the phylogenetic distance and the Bray-Curtis distance of the anti-viral arsenal of diverse species. Each plot corresponds to one species. For each species, the Bray-Curtis distance (dist_BC) of all pairs of anti-viral arsenals was computed as well as the phylogenetic distance (dist_phylo) between all strains. Each line corresponds to a pearson fit of the data.

**
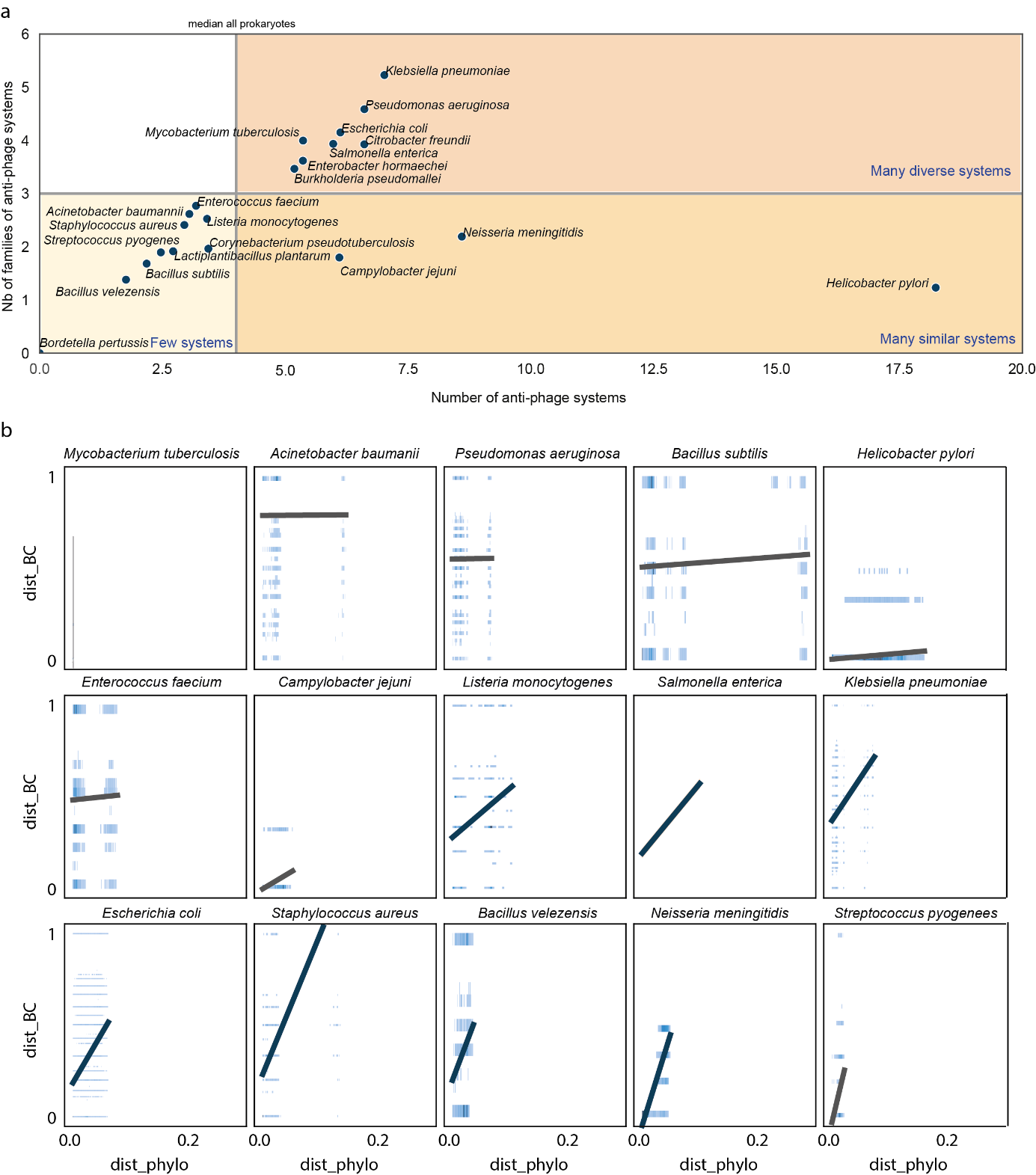
**

**Supplementary Figure 10: Determinants of the anti-viral arsenal of bacterial species**

**a.** Genome size of bacterial species n = 7437 (ranging from 1630 for *Escherichia coli* to 111 for *Corynebacterium pseudotuberculosis*). The box extends from the first quartile (Q1) to the third quartile (Q3) of the data, with a line at the median. The whiskers extend from the box by 1.5x the inter-quartile range (IQR).  **b.** Correlation between the families of antiviral systems and the genome size (Linear regression pearson r=0.65, p-value=0.0014, light pink represents the confidence interval at 95%). **c.** Correlation between the number of antiviral systems and the number of prophages (linear regression r=-0.2 p-value=0.4 light pink represents the confidence interval at 95%). **d.** Correlation between the number of antiviral systems and the genome size (linear regression r=-0.16 p-value=0.5 light pink represents the confidence interval at 95%).


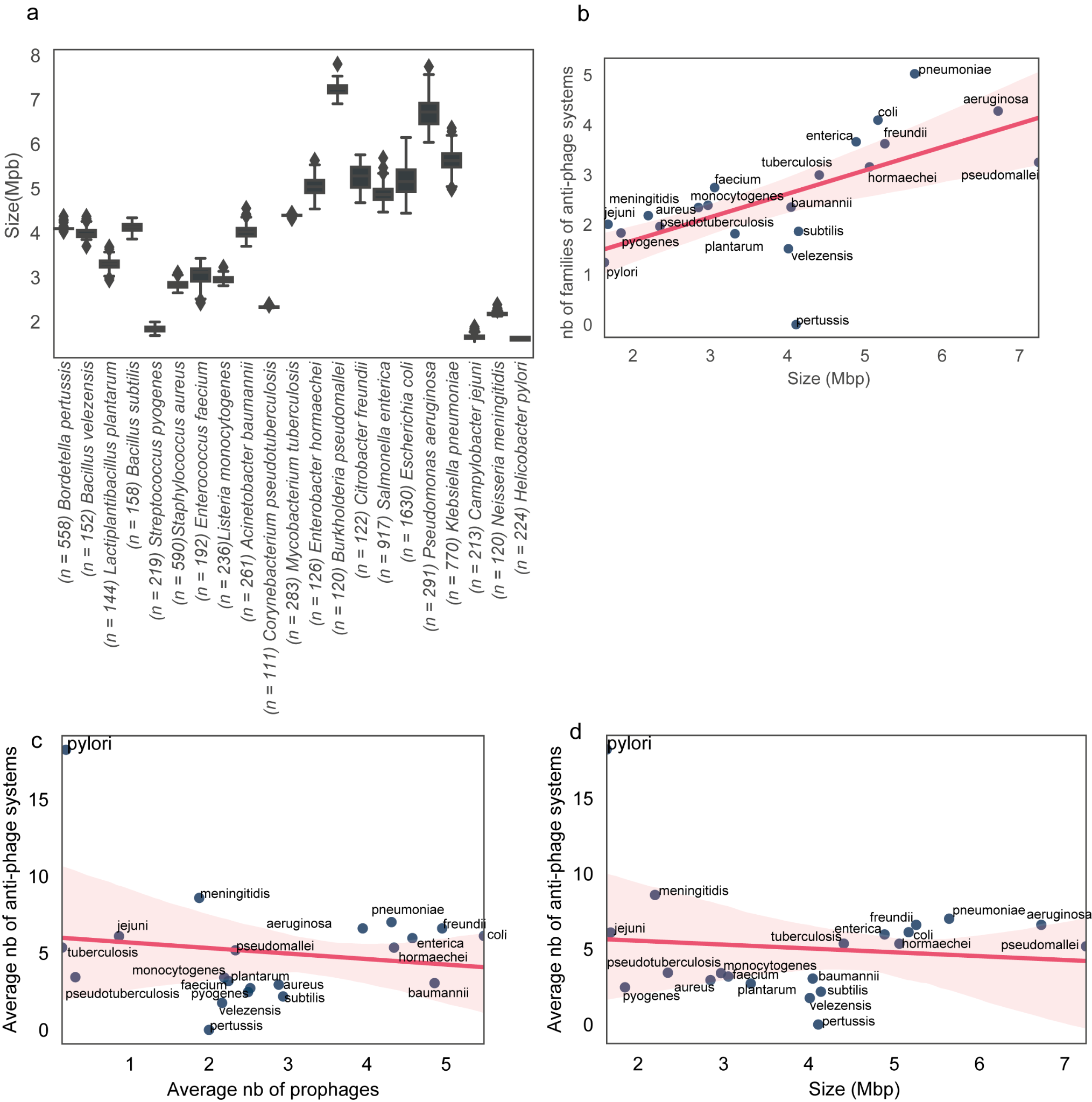

Supplement: Supplementary file 1 — Supplementary Information [file 41467_2022_30269_MOESM1_ESM.docx]
