# Supplementary material for: Systematic and quantitative view of the antiviral arsenal of prokaryotes
Source: Nat Commun. 2022 May 10;13:2561. doi: 10.1038/s41467-022-30269-9 (PMC9090908; doi:10.1038/s41467-022-30269-9)
Supplement: Supplementary file 4 — Description of Additional Supplementary Files [file 41467_2022_30269_MOESM4_ESM.pdf]

Title: Supplementary Data 1:
Description: List of systems, rules and HMM profiles used in DefenseFinder and defense systems mechanisms

Title: Supplementary Data 2:
Description: References for systems, HMM profiles and protein accession numbers used to create HMM profiles.
Systems: List of all systems with the reference paper. HMM: List of all HMM used in DefenseFinder with their name, their systems, their accession number (if not custom), and the GA cut used in DefenseFinder. Protein accession: a table with all the protein accession numbers used in this study, the associated HMM and system and the accession type. (IMG: https://img.jgi.doe.gov/ , NCBI https://www.ncbi.nlm.nih.gov/protein , Patric : https://www.patricbrc.org/ and REBASE http://rebase.neb.com/).

Title: Supplementary Data 3:
Description: Protein Specificity and sensitivity of Doron's systems, DISARM and CBASS
For more details see Methods and Supplementary Figures 1 and 2.

Title: Supplementary Data 4:
Description: RefSeq accession
21 738 genomes accession used in this study.

Title: Supplementary Data 5:
Description: Antiviral systems detected
List of all antiviral systems detected in 21 738 complete genomes. For each system, the table shows system ID (sys_id), NCBI assembly accession (Assembly), replicon, name of the system (syst_name), subsystem (syst_name_type), size of the genome in base pair, begining and end of the system (start_sys and end_sys) and size of the system in base pair (Size_system).

Title: Supplementary Data 6:
Description: Genes involved in anti-phage systems
List of all genes detected in the 21 738 complete genomes that are part of a defense system. Columns are the NCBI assembly accession (ID), gene ID (hit_id), replicon, name of the protein (gene_name), system name (sys_id), the beginning and the end of gene in the genome, system name (syst_name) and subsystem name (syst_name_type).

Title: Supplementary Data 7:
Description: Anti-phage systems per genome

Title: Supplementary Data 8:
Description: Number of genus where each system was detected

Title: Supplementary Data 9:
Description: Number and proportion of genomes without system in all genera with more than 10 genomes.

Title: Supplementary Data 10:
Description: Detection of prophages
